# Methylation Dynamics on 5′-UTR of *DAT1* Gene as a Bio-Marker to Recognize Therapy Success in ADHD Children

**DOI:** 10.3390/children10030584

**Published:** 2023-03-18

**Authors:** Valentina Carpentieri, Gabriella Lambacher, Miriam Troianiello, Mariangela Pucci, Diana Di Pietro, Giovanni Laviola, Claudio D’Addario, Esterina Pascale, Walter Adriani

**Affiliations:** 1 Center for Behavioral Science and Mental Health, Istituto Superiore di Sanità, 00161 Rome, Italy; 2 Faculty of Psychology, Uninettuno University, 00186 Rome, Italy; 3 Servizio Tutela Salute Mentale e Riabilitazione in Età Evolutiva, A.S.L. Roma 6, 00044 Frascati, Italy; 4 Department of Bioscience and Technology for Food, Agriculture and Environment, University of Teramo, 64100 Teramo, Italy; 5 Department of Medical-Surgical Sciences and Biotechnologies, Sapienza University of Rome, 00185 Rome, Italy

**Keywords:** dopamine transporter (*DAT1*) gene, attention-deficit/hyperactivity disorder (ADHD), problematic children, CpGs methylation pattern, methylphenidate (MPH), cognitive-behavioral therapy (CBT)

## Abstract

Attention-deficit/hyperactivity disorder (ADHD), a neuropsychiatric condition characterized by inattention, hyperactivity, and impulsivity, afflicts 5% of children worldwide. Each ADHD patient presents with individual cognitive and motivational peculiarities. Furthermore, choice of appropriate therapy is still up to clinicians, who express somewhat qualitative advice on whether a child is being successfully cured or not: it would be more appropriate to use an objective biomarker to indicate whether a treatment led to benefits or not. The aim of our work is to search for such clinical biomarkers. We recruited 60 ADHD kids; psychopathological scales were administered at recruitment and after six weeks of therapy. Out of such a cohort of ADHD children, we rigorously extracted two specific subgroups; regardless of the initial severity of their disease, we compared those who obtained the largest improvement (ΔCGAS > 5) vs. those who were still characterized by a severe condition (CGAS < 40). After such a therapy, methylation levels of DNA extracted from buccal swabs were measured in the 5′-UTR of the DAT1 gene. CpGs 3 and 5 displayed, in relation to the other CpGs, a particular symmetrical pattern; for “improving” ADHD children, they were methylated together with CpG 2 and CpG 6; instead, for “severe” ADHD children, they accompanied a methylated CpG 1. These specific patterns of methylation could be used as objective molecular biomarkers of successful cures, establishing if a certain therapy is akin to a given patient (personalized medicine). Present data support the use of post-therapy molecular data obtained with non-invasive techniques.

## 1. Introduction

The most recent studies by our group focused on biomarkers useful as predictors of therapy efficacy; this study in particular focused on patients affected by attention-deficit/hyperactivity disorder (ADHD), a neuropsychiatric condition characterized by inattention, hyperactivity, and impulsivity [1]. It is estimated that around 5% of children and 2.5% of adults are affected by ADHD worldwide [2].

ADHD symptoms are complex and heterogeneous [3]. Affected individuals differ from each other on cognitive and motivational profiles and on tagged brain structures. Etiology also varies in terms of genetic risks as well as environmental exposures [4]. Recognizing such a heterogeneity could lead to the adoption of precision medicine; the trend in recent years is indeed to recognize and personalize individual treatments in order to improve cognitive and motivational processes in a specific ADHD individual [5]. The neuropsychological sub-categorization of the ADHD population (i.e., inattentive vs. impulsive) has been proposed to facilitate this type of approach [6].

Many studies on ADHD take into consideration a well-known human gene such as SLC6A3 also called Dopamine Transporter (*DAT1*). The *DAT1* gene encodes a member of the sodium- and chloride-dependent neurotransmitter transporter family; a dopamine (DA) active transporter which is crucial for DA reuptake. Differential expression of *DAT1* gene, with consequent alterations of basal DA, has been proposed to explain ADHD pathogenesis, at least in part [7].

In 2014, van Mil and colleagues sought to establish whether methylation levels observed at birth in neuronal genes were associated with the onset of ADHD symptoms at the age of 6 years [7]. Analysis of 11 genomic regions showed that lower DNA methylation levels were associated with more severe symptoms in ADHD individuals. However, the authors were unable to distinguish whether genetic transmission and/or unknown environmental factors underlie this association [7]. Similarly, Walton and colleagues analyzed a sample of 817 ADHD children and identified 13 genomic locations, where DNA methylation level was significantly predictive of ADHD symptom trajectories between 7 and 15 years of age [8]. In 2017, Ding and colleagues demonstrated that the methylation profile in the DAT1 gene is modulated in response to methylphenidate (MPH) treatment, along with its efficacy on hyperactive–impulsive symptoms in ADHD [9].

Our own studies on ADHD take into consideration the methylation pattern in the 5′-UTR of the DAT1 gene [10]. The correlation between methylation patterns in the 5′-UTR of the DAT1 gene and psychopathological condition or addiction has been widely used in previous studies [11,12,13]. In our last papers [14,15], we investigated whether the methylation pattern in the DAT1 gene (including the opposed DNA strand) can be influenced by the allelic genotype at 3′-UTR. Namely we drew comparisons in 9/9 or 10/10 DAT1 homozygous children or in heterozygous ones, born from 9/10 heterozygous mothers (and 10/10 fathers) or 9/10 heterozygous fathers (and 10/10 mothers). We also addressed whether these factors correlate with a given psychopathological phenotype [11].

This recent sample in particular was analyzed with a correlation approach [14] that was proposed to find unsuspected insights [15]. We therefore sought to apply the same approach to quite old data [10,14]; ADHD children aged 6–12 had undergone psychopathological scales for ADHD at recruitment, then underwent a cognitive-behavioral therapy (CBT) or MPH [16,17] based on the severity of their disease. Six weeks after the onset of therapeutic efficacy, all the scales were repeated to define success or not in an objective, unbiased way.

Taking the two extreme tails of patients’ distribution, we ended up with two main subgroups to be analyzed: “ADHD improving” children, who had a greatest improvement after therapy regardless of the severity of their initial condition, and “severe ADHD” children, who got the highest severity score in the same psychopathological scales and maintained it despite therapy. The improving subgroup includes both treatments, namely ADHD children who improve with MPH as well as those who do so with CBT. In fact, some authors specify that both therapies are valid for the recovery from ADHD [18,19].

The final goal of this line of research was to identify a possible novel biomarker, to early recognize therapeutic efficacy over psychopathological vulnerability. The epigenetic changes that occur on the *DAT1* gene ended up as possible biomarkers that can help the clinical validation of a successful therapy. As mentioned above, such kind of studies are relevant for tailored interventions, namely what is now called personalized medicine.

## 2. Methods

### 2.1. Recruitment of the Sample

The sample was made up of 60 children (6–12 years old) with a diagnosis of ADHD formulated (from April 2010 to March 2012) by the Child Psychiatry Unit of Tor Vergata University according to DSM-IV-TR criteria [20]; a medical examination was performed to diagnose the presence of comorbid disorders. Fifteen children with a normal condition were used as controls. Children who had one or more psychiatric comorbidities (i.e., obsessive-compulsive disorder, conduct disorder, depression, bipolar disorder, and psychosis) were excluded from the study. The presence of the aforementioned comorbidities was assessed via the K-SADS/PL diagnostic interview [21].

This study was approved by the Ethical Committee of ISS, Prot. CE-ISS 09/270 of 15 July 2009 (as study ended on 15 July 2012, obligations to keep raw data ended on 15 July 2022); the parents signed written informed consent for their child well before participating. The Code of Ethics of the World Medical Association (“Declaration of Helsinki”), as printed in the British Medical Journal (18 July 1964), were fully respected.

Once the children were recruited as patients, direct observations and clinical questionnaires were administered. The Children’s Global Assessment Scale (CGAS) valued the severity of psychiatric disorder [22]. The CGAS scores range between 1 and 100, with a >70 value indicating a normal condition for children aged 4–16 years. The presence of ADHD subtypes (inattentive, hyperactive-impulsive, and combined types) was diagnosed using the Conners’ Parent Rating Scale [23].

According to current guidelines and practice in Italy, the more severe ADHD children were assigned to MPH therapy (CGAS < 40). Less severe ADHD children were assigned to CBT (41 < CGAS < 55); in case of unsuccessful CBT, patients were moved to MPH. Both therapies lasted for six weeks at least after reaching optimal dosage and/or compliance, after which all scales were repeated. The Δscores (i.e., value after therapy minus value at recruitment) were calculated to assess the extent by which children’s symptoms either improved or did not respond to therapy.

After these six weeks of therapy, during a follow-up of patients, a sample was collected for each patient using a buccal swab (Swab Pack, Isohelix, Harrietsham, Kent, UK), a non-invasive technique for sampling DNA.

### 2.2. Building Up of Two Subgroups

Starting from the initial sample of sixty ADHD patients, two specific subgroups of children were rigorously selected. The first subgroup (*n* = 9; 8 males and 1 female) was composed of patients who were still “severe” (i.e., those still characterized by a CGAS < 40 when the scoring was repeated after the six-week treatment). The second subgroup (n = 10; 8 males and 2 females) consisted of children who, regardless of the initial severity of their disease and regardless of which therapy, managed to obtain the largest improvement after therapy based on the calculation of Δscores (ΔCGAS >> 5). This subgroup of children was termed the “tail” of most “improving” subjects.

The two subgroups thus selected represented the two extremes of a Gaussian curve, in the middle of which there were all other patients who responded less clearly or more slowly to therapy. All these remaining patients, who were present in the initial sample [10], were not considered presently. These patients included (a) those who, refusing any drug, had been treated with CBT but failed to respond to therapy; (b) those who decided to drop out; and (c) MPH-resistant children who were eventually moved to other drugs (e.g., atomoxetine). In our clinical experience, there may be up to one-third of patients who do not achieve any consistent improvement regardless of MPH therapy.

### 2.3. Measurement of 5′-UTR CpG’s Methylation Level

DNA was isolated using the Buccal-prep Plus DNA isolation kit (Isohelix) according to the manufacturer’s protocol: 0.5 micrograms of DNA from each subject was treated with bisulfite using a DNA methylation kit (Zymo Research), and the levels of methylation were determined as previously described [10,11].

**Figure 1:**
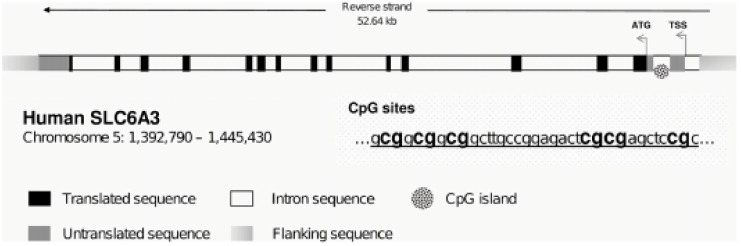
The human SLC6A3 gene or *DAT1* is represented. The abbreviations TSS and ATG represent the transcription start site and the translation start site, respectively. CpG island is located in the 5′-UTR of the gene and contains the five CpG sites (in bold) we studied. The underlined region is reported from +712 to +746 relative to TSS; the first “cytosine-P-guanine” is positioned at 1,444,717 (at +713) and 1,444,716 (at +714) on the reverse strand of chromosome 5.

All the six CpG sites being studied are located on chromosome 5 (1,444,685–1,444,717; first intron of *DAT1* at +713 from TSS) and are shown in the following sequence: ^1^CGG ^2^CGG ^3^CGG CTT GCC GGA GAC T^5^CG ^6^CGA GCT C^7^CG. The first three CpG loci (^1^CGG ^2^CGG ^3^CGG) represent the first motif; CpG loci 5 and 6 (^5^CG ^6^CG) were the second motif we studied (Figure 1).

### 2.4. Linear Correlation between CpGs: Patterns of Methylation

The purpose of this new and pioneering calculation was to ascertain whether the 5′-UTR of the *DAT1* gene has specific dynamics of methylated CpGs in a specific clinical condition. Other methods aim usually just to measure general low or high methylation levels of the entire gene (introns, 5′-UTR, or promoter regions). Our interest instead was to investigate whether the pattern of methylation can be precise (i.e., with one CpG rising when another CpG falling) at the level of specific CpG motifs present in the *DAT1* 5′-UTR region [10,14].

The very simple, although sophisticated, approach (that we present here) evaluated linear correlations between singles and pairs of CpGs at the *DAT1* 5′-UTR. The pair is made up by multiplying raw levels of two CpGs together. In fact, since methylation is presented as a percentage, their product represents the probability that such two events occur simultaneously. Therefore, the pairs denote the probability that two CpGs are simultaneously (de)methylated at a given time. In this way, we aimed at looking to the most probable pattern of “getting” (de)methylated CpGs.

In particular, we calculated all these possible correlations using Pearson’s coefficient of correlation. A single CpG can be methylated (M) or demethylated (100 − M = D). The same happens in the pair of multiplied CpGs in which each component of the product can be either methylated (M) or demethylated (D). A positive or negative correlation was significant (*p* < 0.05) only for *r* > ± 0.82. For *r* < 0.33, the correlation was not significant [14]. All math steps were calculated using custom-made scripts in MATLAB.

All the significant correlations were then compared between the two subgroups, and all those that differentiated the two subgroups from each other were included in the results. When a significant positive correlation was found on the same triad of CpGs in both groups, this was excluded from the results as not interesting for the experiment.

## 3. Results

The severe subjects were characterized by:

A significant positive correlation (*r* = 0.93) between M1 and D2M3; a significant positive correlation (*r* = 0.92) between M1 and D6M3; a significant positive correlation (*r* = 0.88) between M3 and D2M1; a significant positive correlation (*r* = 0.90) between M3 and D6M1; a significant positive correlation (*r* = 0.86) between M1 and D2M5; a significant positive correlation (*r* = 0.83) between M5 and D2M1; a significant positive correlation (*r* = 0.95) between M3 and D2M5; a significant positive correlation (*r* = 0.97) between M3 and D6M5; a significant positive correlation (*r* = 0.97) between M5 and D2M3; a significant positive correlation (*r* = 0.96) between M5 and D6M3; a significant positive correlation (*r* = 0.97) between M3 and M1M5; and a significant positive correlation (*r* = 0.91) between M5 and M1M3.

All the results can be viewed in Table 1.

The improving subjects were characterized by:

A significant positive correlation (*r* = 0.85) between M3 and M2M5; a significant positive correlation (*r* = 0.85) between M3 and M2M6; a significant positive correlation (*r* = 0.82) between M3 and D1M5; and a significant positive correlation (*r* = 0.87) between M3 and M5M6.

All the results can be viewed in Table 1 as well.

## 4. Discussion

In the original study by our group [10], we observed a portion of ADHD children responding perfectly to the CBT alone, despite the fact they were facing (at least in some cases) a serious condition of the disease. On the contrary, even children with less severe ADHD might not respond at all, even to the MPH therapy. Apparently, an initial clinical severity of the diagnosis is not a guarantee that either MPH or CBT will be an effective therapy for a given patient. To get potential insights, we re-analysed our own raw dataset using a multiple correlation approach [11].

**Figure 2:**
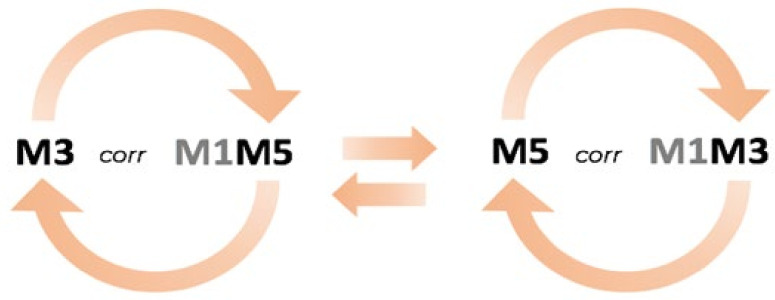
Example of “seesaw”-like behaviour. Methylated CpG 3 (M3) found as a single CpG is correlated with the multiplied pair of CpGs 1*5 (M1M5). In passing to the right, CpG 3 is interchanged with CpG 5; the latter now gets correlated as a single (M5) instead of CpG 3, and the former will now be found within the multiplied pair. CpG 1 remains to complete the triplet (M1M3).

**Figure 3:**
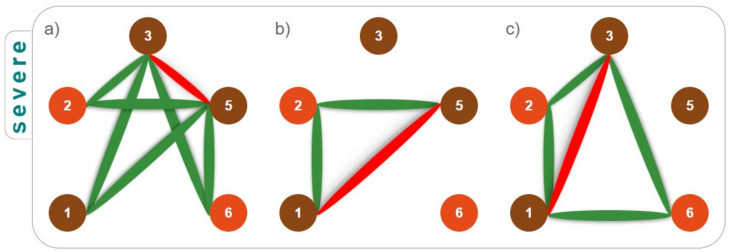
Pattern of methylations in severe ADHD subjects. Methylated CpGs are represented with a brown circle while the demethylated ones are depicted in orange. The red connector links the two CpGs that are behaving like a “seesaw”. The triplet is then completed with a third CpG, always found within the multiplied pair, which is reached by the green connectors. In severe ADHD subjects, CpGs 3 and 5 (**a**), CpGs 1 and 5 (**b**), and CpGs 1 and 3 (**c**) compose such seesaws.

As seen in Table 1 line a1, the methylated CpG 3 is found as a single correlated with the methylated CpG 5, which is multiplied to the demethylated CpG 1; in the row immediately below (Table 1, line a2), where the methylated CpGs 3 and 5 interchanged in particular, we draw the attention to CpG 5 correlating as a single while CpG 3 now found within the pair, multiplied with CpG 1. We propose to define this particular correlation pattern as “seesaw” (Figure 2); these methylation patterns are graphed with red connectors that link interchanged CpGs. To complete the triplet of CpG 3/CpG 5 “seesaw”, we found not only the methylated CpGs 1 (within the pair), but also demethylated CpG 2 and demethylated CpG 6 (Figure 3a).

Severe children are also characterized by two additional “seesaws”: the first between CpGs 1 and 5 (Table 1, lines a7 and a8) and the second between CpGs 1 and 3 (Table 1, lines a9–a12). To complete the triplet of the CpG 1/CpG 5 “seesaw”, we found a demethylated CpG 2 inside the multiplied couple (Figure 3b); to complete the triplet of CpG 1/CpG 3 “seesaw”, we can have both the CpG 2 and CpG 6 always demethylated (Figure 3c, green connectors).

**Figure 4:**
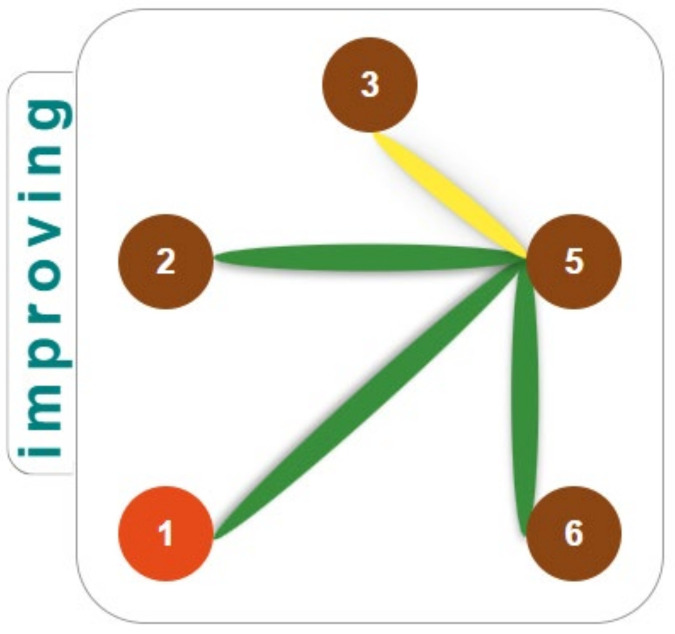
Patterns of methylation found in improving ADHD subjects. CpGs 3 and 5 make an incomplete “seesaw” (yellow connectors), always locating as a single and within the multiplied pair, respectively. The triplets are completed with the CpGs 2 and 6 always multiplied with CpG 5 (green connectors).

In the improving subgroup, the patterns of methylation is definitely different as we cannot find specific CpGs that pass from being correlated as a single to being correlated as multiplied. Rather, a CpG 3 always stays as a single correlated (Figure 4, yellow connector), with multiplied pairs that always include CpG 5, associated with CpGs 2 and 6 (always methylated), and with a CpG 1 (always demethylated). This pattern is strikingly more simple (Figure 4).

Therefore, subgroups of children are uniquely characterized by the methylated CpG 1 in the severe subgroup on the one hand and methylated CpGs 2 and 6 in the improving subgroup on the other side [10]. It can be seen that, for the severe subgroup in particular, only CpGs 1, 3, and 5 are methylated together and present a methylation pattern consistent with previous data [15]. On the contrary, in the improving subgroup, CpGs 2, 3, 5, and 6 are methylated while CpG 1 is always demethylated.

Noteworthy, the CpGs 3/5 pass from the same destiny of CpG 1 in the former case to the same destiny of CpGs 2/6 in the latter case. In addition, the five CpGs were all actively engaged in a symmetric seesaw pattern in the severe subgroup; conversely, this symmetry was somewhat lost (in that CpGs 3 and 5 cease to interchange) within the improving subgroup. To uniquely characterize the two subgroups, it is evident that CpGs 3 and 5 cannot be useful; both present the same methylation “dynamics” (not levels).

### 4.1. Limitations

The present work is based on two selected subgroups composed of quite a limited number of subjects. In creating specific subgroups of patients from a larger initial sample, small samples are often obtained that are still useful for the aims of a study if they are extremely specific. However, a further in-depth study should take an even larger initial sample of ADHD children, in order to obtain larger and even more specific subgroups of greatly ‘improved’ and greatly ‘severe’ ADHD children.

It would also be interesting to further separate children who improved into two: (a) patients with mild/moderate ADHD and improved after being treated with CBT vs. (b) those who had a severe condition and improved after treatment with MPH. However, the clinical setting is heterogeneous by definition, in that there were (and always there will be) mild/moderate ADHD patients needing to deal with MPH as well as those who had a severe condition yet improving after a careful CBT treatment.

Our sample also lacks any children characterized by mild/moderate ADHD and who did not respond to CBT. In fact, among all the children who were initially given this non-pharmacological therapy, the few who did not get any recovery either had to be moved to MPH prescription or abandoned the therapy and, therefore, the study as well. With a larger sample of ADHD children, it may also become possible to study such a subset. However, further expanding the number of individuals to-be-recruited, solely to analyze the therapies (i.e., subtle putative differences in methylation patterns after recovery from one therapy rather than another), might not bring any valuable benefit in terms of the exploitation of present applications. The declared goal of our study was to obtain a healing-predictor biomarker regardless of the therapy used. Ultimately, an increased recruitment of ADHD children, whose number should at least double, would entail doubling the required time-window (three years in our hands) or doubling the involved Clinical Centers, which may have an ineligible cost/benefit analysis.

In addition, unfortunately, methylation levels were measured only once following six weeks of CBT or MPH therapy. It would be advisable to repeat the approach exploiting multiple measures, namely before, in between, and after therapy, just to confirm the dynamic pattern we found as a reliable biomarker of improvement. The clinical experiment should be also replicated by verifying whether other treatments for ADHD (and their variable rates of therapeutic success) do produce the same changes within methylation patterns of the DAT1 5′-UTR.

When searching for a biomarker to recognize the success of a therapy, the sampling of DNA should be non-invasive, even more so when patients are children. In many recent studies, buccal swabs or saliva are used to analyze the methylation of genes involved in psychiatric diseases. Recent studies demonstrate that DNA methylation levels in epithelial cells and in the brain are very similar [24,25]. This makes buccal-swab sampling of saliva a good non-invasive method for analyzing methylation levels in developmental behavioral diseases [26,27].

### 4.2. Future Perspective

In general, the 5′-UTR of DAT1 gene has the characteristic feature of possessing palindromic sequences, which may turn out to provide even further clinical biomarkers. For instance, within 50 bp downward from the sequences we studied presently, there are two other interesting motifs: +747 CACCCGACG +755 and +799 CGTCGGGTG +807 (which would be CpGs 8, 9, 16, 17 had we continued with numeration). It is apparent that the second (CpGs 16–17) motif is exactly the same sequence that is complementary to the first (CpGs 8–9) motif if going backward, and identical to the first (CpG 8-COS/9-COS) motif on the inverted-direction noncoding DNA (complementary opposite strand, or COS) (Figure 1). As such, the first motif on the main (gene) strand may alternatively bind to either an identical sequence, one being the original found on the COS or another found on the very same coding-strand surroundings, fifty bp downwards, if this coding strand bends and comes back antiparallel after a loop.

These traits, with alternative possibility, could very well undergo somewhat coordinated actions; when the double-helix opens, the CpG 16/17 may loop back and, antiparallel, bind to the CpG 8/9 trait; then, such loops may be proposed to serve as a checkpoint to keep the two strands separated (or, to start separating) when transcription shall occur.

### 4.3. Conclusions

The present methylation patterns are exclusive of specific and well-characterized subgroups. Our punctual methylation patterns were found in those individuals either who have greatly recovered or who have not undergone any improvement after any therapy. In very recent years, the personalized medicine is becoming more and more popular [28]; indeed, each individual may respond differently to a certain therapy, benefiting or not from it.

A large number of children and young adults with ADHD are taking a psychostimulant drug for a long time without benefiting from it, and this is clearly harmful [29,30]. The topic concerning personalized medicine does not refer only to ADHD but also to countless pathologies [31,32,33]. Therefore, research is warranted to validate measurable endpoints that might potentially be used as clinical biomarkers. Furthermore, whether or not a patient is being cured effectively can be established in a currently qualitative way; no objective molecular data exist yet that can reliably establish how much the therapy has benefited to the patient.

We hypothesized the following objective biomarkers: (a) the methylation of CpG 2 and CpG 6 could objectively demonstrate the successful caring of the ADHD child; (b) the methylation of CpG 1 could be used as biomarker of a severe ADHD diagnosis who will not improve. As a whole, a biomarker for (un)favorable prognosis may be served by (anti)parallel dynamics between CpGs 3/5 and CpGs 2/6. This kind of evidence could indicate, perhaps objectively, if the chosen therapy was or was not the most suitable for that particular ADHD child.

## Figures and Tables

**Table 1:** Pearson’s *r* correlations. Only significant items are shown.

(a) Severe Subjects	
M3 M1M5	0.97
M5 M1M3	0.91
M3 D2M5	0.95
M5 D2M3	0.97
M3 D6M5	0.97
M5 D6M3	0.97
M1 D2M5	0.86
M5 D2M1	0.83
M1 D2M3	0.93
M3 D2M1	0.88
M1 D6M3	0.92
M3 D6M5	0.9
	*p* < 0.05
**(b) Improving Subjects**	
M3 M5D1	0.82
M3 M5M2	0.85
M3 M5M6	0.87
M3 M2M6	0.85
	*p* < 0.05

## Data Availability

Not applicable. Obligation to keep original raw data stored expired as of 15 July 2022.
